# Interactions between Soil Habitat and Geographic Range Location Affect Plant Fitness

**DOI:** 10.1371/journal.pone.0036015

**Published:** 2012-05-17

**Authors:** John Stanton-Geddes, Ruth G. Shaw, Peter Tiffin

**Affiliations:** 1 Department of Ecology, Evolution and Behavior, University of Minnesota, St. Paul, Minnesota, United States of America; 2 Department of Plant Biology, University of Minnesota, St. Paul, Minnesota, United States of America; Norwegian University of Science and Technology, Norway

## Abstract

Populations are often found on different habitats at different geographic locations. This habitat shift may be due to biased dispersal, physiological tolerances or biotic interactions. To explore how fitness of the native plant *Chamaecrista fasciculata* depends on habitat within, at and beyond its range edge, we planted seeds from five populations in two soil substrates at these geographic locations. We found that with reduced competition, lifetime fitness was always greater or equivalent in one habitat type, loam soils, though early-season survival was greater on sand soils. At the range edge, natural populations are typically found on sand soil habitats, which are also less competitive environments. Early-season survival and fitness differed among source populations, and when transplanted beyond the range edge, range edge populations had greater fitness than interior populations. Our results indicate that even when the optimal soil substrate for a species does not change with geographic range location, the realized niche of a species may be restricted to sub-optimal habitats at the range edge because of the combined effects of differences in abiotic and biotic effects (e.g. competitors) between substrates.

## Introduction

Populations at different locations often occupy different habitats [1]–[5]. This change in habitat could be because these are the sites that receive migrants, or because these are the sites where migrants successfully establish. While animal migrants often select their habitats [3], [5], in sessile species the habitat is more likely to select the migrants that survive and establish stable populations [6]. At the edge of a species' range, where individuals are likely to be at the limits of their physiological tolerances [7], [8], the habitat typical of the majority of the species' geographic range may not be suitable. For example, at its northern limit in Quebec, eastern hemlock (*Tsuga canadensis*) is found more often on habitats with northern and western slopes than within its range [2]. In the context of climate change, such shifts in habitat at range edges could be indicative of the habitats in which leading edge populations establish as they shift their distributions, or where trailing edge populations may persist within the range. However, few studies have examined how fitness depends on habitat type at range edges.

Soil characteristics are key aspects of the habitat which are spatially variable. Because soils vary in nutrient levels, water diffusion, metal concentrations [9], and because they support different biotic communities [10]–[12] they can have strong effects on plant growth and fitness [13], [14]. Further, the environment a plant experiences is partially dependent on interactions between soil substrate and climate. For example, at *Clarkia xantiana*'s eastern range edge in southern California, water availability is much lower than expected based on precipitation because of a change in soil type [15]. Thus, the niche in which individuals establish and reproduce may differ within, at and beyond the current range edge.

Where populations establish will also depend on their origin. Populations are often locally adapted [16], [17] to factors including climate [18]–[21], competitors [19], [22], natural enemies [23] and soil [24]. If populations at the range edge have already adapted to marginal conditions, individuals from these populations may be most likely to generate new populations beyond the current distribution. However, peripheral populations are often [8], [25] but not always [26] small, and thus adaptation to local conditions may be constrained by drift or gene flow from interior populations [27] under some but not all conditions [28], [29].

**Figure 1:**
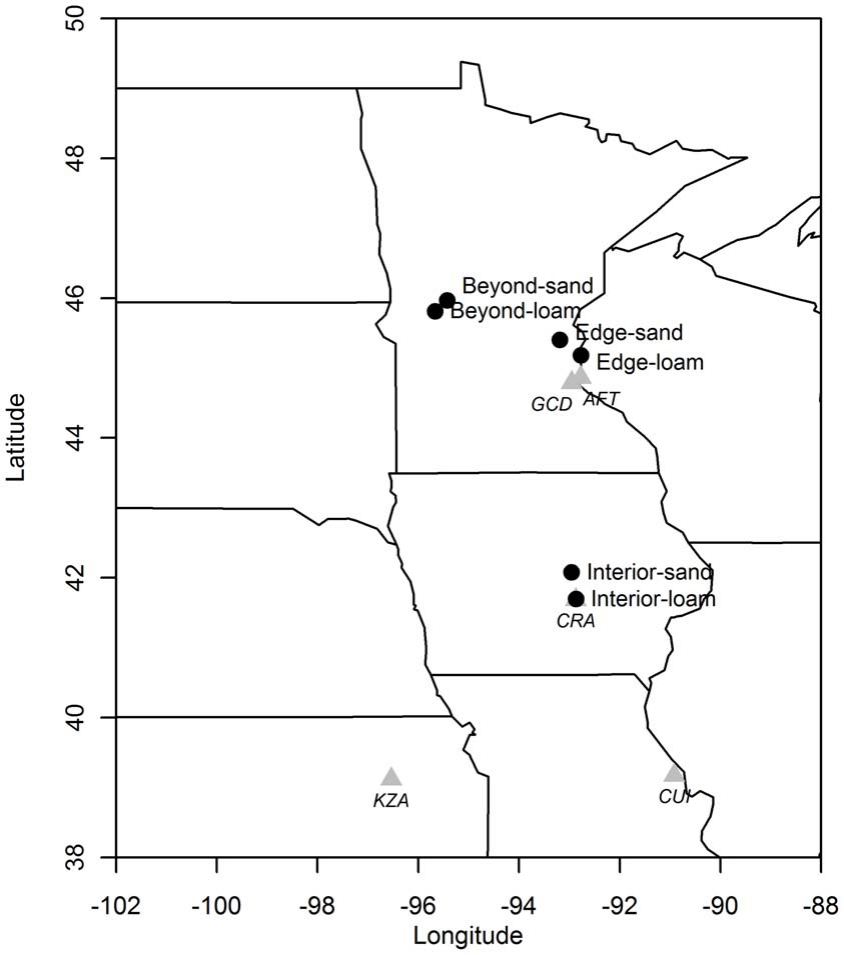
Map of seed source populations and transplant common garden locations. Seed source populations (Table S1) are marked by triangles (KZA: Konza Prairie Biological Station; CUI: Cuivre River State Park, CRA: Conard Environmental Research Area; GCD: Grey Cloud Dunes Scientific and Natural Area; AFT: Afton State Park). Common garden locations (Table S2) are marked by black circles. The dotted line is the approximate range edge in this region, based on USDA Plants Database county level information.

In this study, we used the native annual legume *Chamaecrista fasciculata* to investigate; (1) the extent to which plant fitness is influenced by habitat at different geographic range locations, and (2) variation in fitness among populations from different geographic locations when transplanted beyond the range edge. We accomplished these objectives by planting seed from five populations on two soil substrates, loam and sand, within the species' range, at its current range edge, and beyond the range edge (Fig. 1). By planting field-collected seed, we are evaluating the potential for colonists to establish after a simulated dispersal event. We recorded the survival and reproduction of each individual, and jointly analyzed the data using aster models which integrate multiple components of life history (Fig. S1) in a single analysis [30], [31]. We chose the two soil types because although *C. fasciculata* is found on soils ranging from sand to clay within its range [32], it is primarily found on sand soils at its range edge [33], [34]. Because our focus was on the effects of soil type on fitness, and competitors vary considerably between habitat types and regions, we minimized differences in above-ground vegetation via herbicide application and mowing prior to planting at each site. We are thus determining the fundamental niche immediately available to colonists beyond the range edge.

## Results

**Figure 2:**
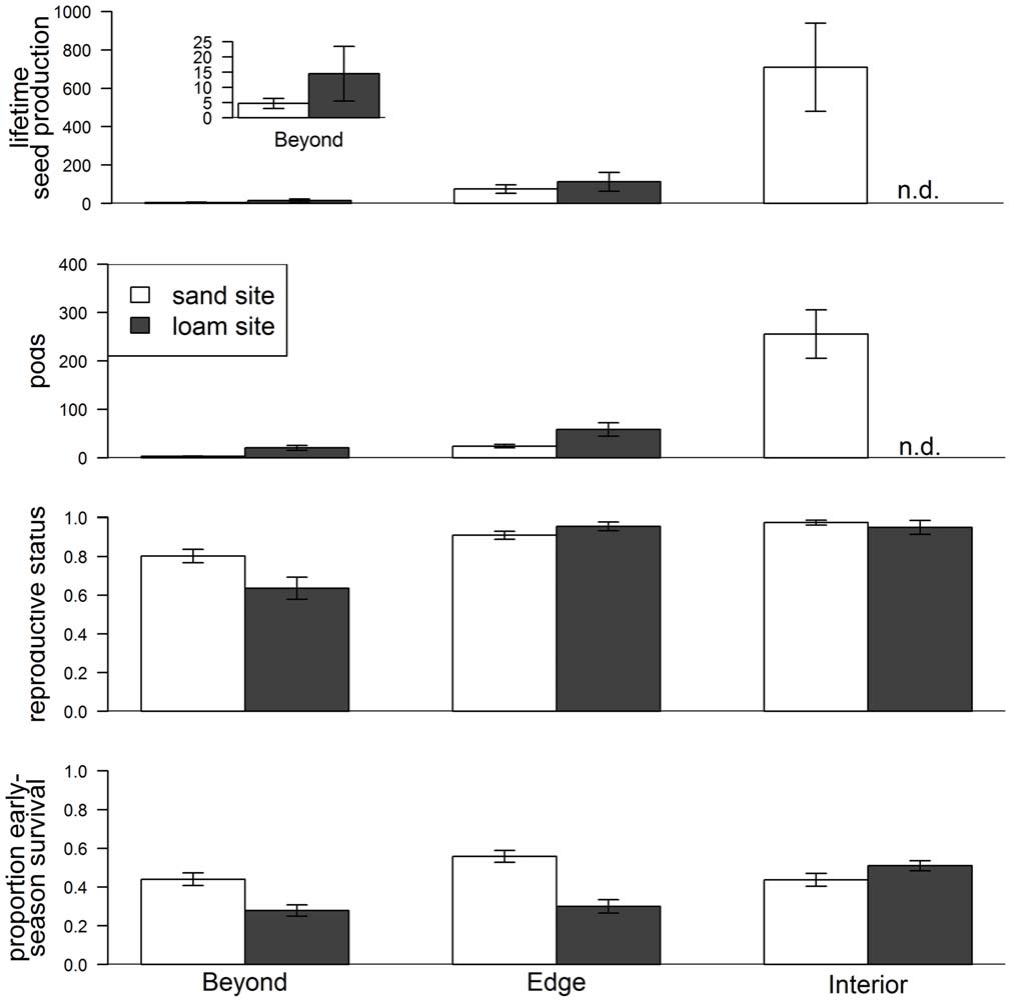
Maximum likelihood estimates of life history stages at each site in each region. Estimates from the best-fit aster model of proportion early-season survival, reproductive status (whether a plant reproduced or not) given survival, pods produced per plant given reproduction, and lifetime seed production, integrating across the previous stages. Bars represent standard errors. The inset plot shows lifetime seed production for the sites in the Beyond region. At the interior-loam site, the values for survival and reproductive status come from the observed data as all plants were destroyed before end of season data was collected (n.d.) so this site was not included in the aster analysis.

Lifetime seed production, considering all populations together, was significantly higher in the interior (∼710 seeds produced for each seed planted) than in the edge (∼94 seeds/seed planted) or beyond edge (∼10 seeds/seed planted) regions (Table 1, Fig. 2). There was a significant interaction between transplant region and soil type (Table 1). At the range edge and beyond edge, seed production was ∼49% and 212% greater at the loam site than at the sand site, respectively. In the interior region, we were not able to measure seed production as deer destroyed all plants at the interior-loam site before the end of the season. Although more seeds were produced at the loam than sand habitats in the edge and beyond edge regions, early-season survival was greater at the sand habitat in both locations (Fig. 2). By contrast, in the interior region, early-season survival was slightly greater at the loam than the sand habitat (Fig. 2). Reproductive status (whether a plant produced any pods given survival) was also greater at the sand than the loam site beyond the range edge, and roughly equivalent at the edge and interior regions (Fig. 2). However, for those plants that produced seeds, seed pod production was much greater at the loam site than the sand site in both the edge and beyond edge regions (Fig. 2).

**Table 1:** Summary of aster model comparisons to test for effects of region and habitat on *C. fasciculata* lifetime seed production.

Model	Model d.f	Model deviance	Test d.f.	Test deviance	*P*-value
Full	45	50430	-	-	-
Block	30	50450	15	21.4	0.13
Region × Habitat	40	50881	5	458	<0.0001
Region	30	54376	10	3542	<0.0001
Habitat	35	51299	5	422	<0.0001

The full model included fixed effects for block, region, habitat and region × habitat with lifetime seed production, consisting of multiple life history stages (Fig. S1), as the response. Likelihood ratio tests were used to compare the fit of the full model to reduced models that sequentially dropped terms. Analysis of deviance (−2 log likelihood) and χ^2^ *P*–values for each model test are listed. The block and the interaction term were tested against the full model, while the region and soil terms were tested against the model without the interaction term.

**Table 2:** Summary of aster model comparisons to test for effects of population, region and soil and all interactions on *C. fasciculata* lifetime seedpod production.

Model	Model d.f	Model deviance	Test d.f.	Test deviance	*P*-value
Full	93	42649	-	-	-
Block	78	42666	15	16.8	0.33
Pop × Region	69	42738	24	88.8	<0.0001
Pop × Soil	81	42677	12	28.7	0.005
Region × Soil	88	42738	5	360.8	<0.0001
Without interactions	46	43177	-	-	-
Pop@esurv	40	43243	2	61.1	<0.0001
Pop@pod	42	43182	2	1.1	0.59
Pop@seed	44	43181	2	4.0	0.14
Region	32	46292	10	3109	<0.0001
Soil	37	43672	5	490.1	<0.0001

The full model included fixed effects for block, region, habitat, population and all interactions, with lifetime seed production, consisting of multiple life history stages (Fig. S1), as the response. The effect of population was tested at multiple life history stages (@seeds, @ pods and early-season survival (@esurv). Likelihood ratio tests were used to compare the fit of the full model to reduced models that sequentially dropped terms. Analysis of deviance (−2 log likelihood) and χ^2^ *P*-values for each model test are listed. The block and the interaction terms are tested against the full model, while the population, region and soil terms were tested against the model without interactions. Only populations planted at all sites (CRA, GCD, KZA) were included in this analysis.

**Figure 3:**
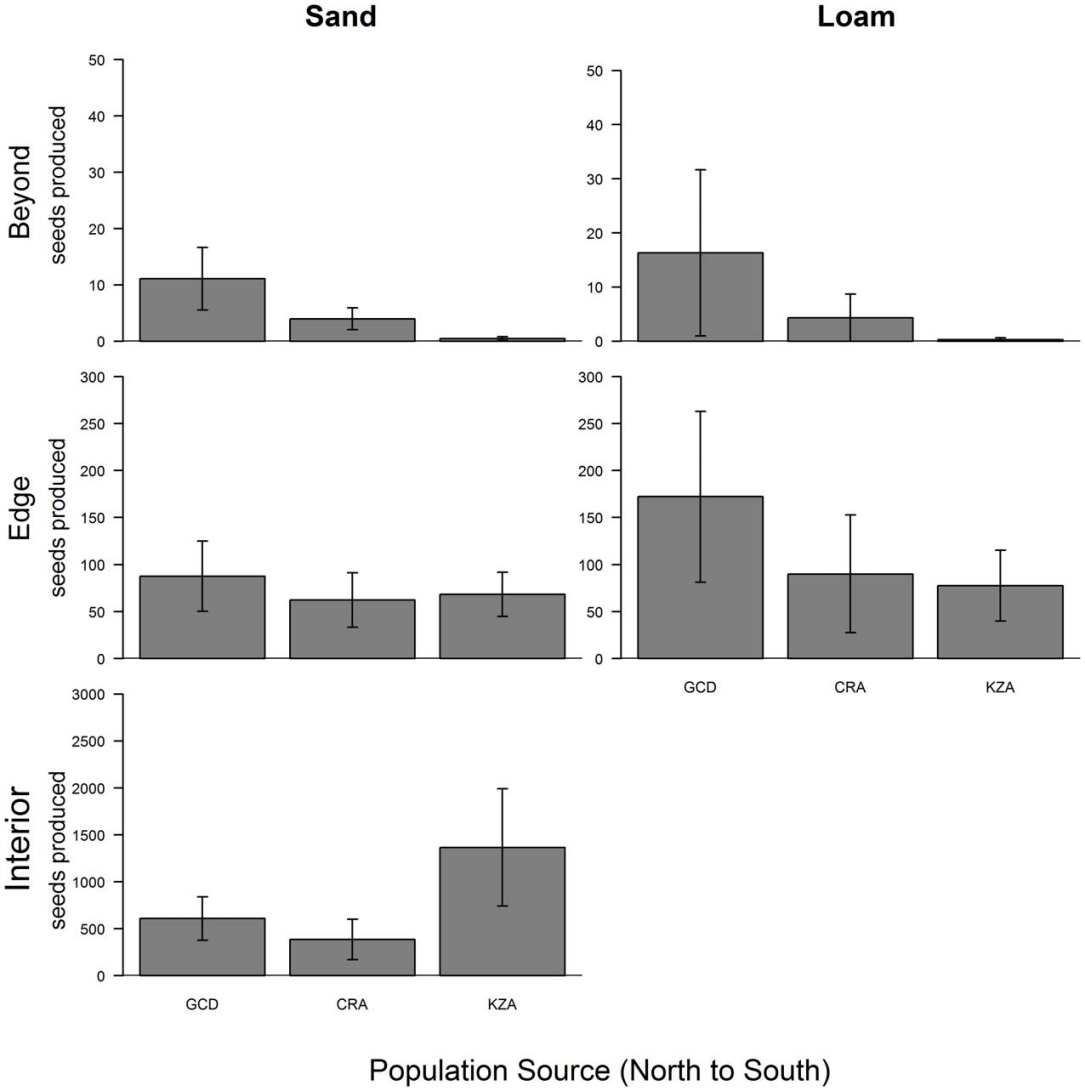
Maximum likelihood estimates of lifetime seed production for each population planted at each site. Unconditional estimates of lifetime seed production, integrating across the previous stages, from the best-fit aster model. Bars represent standard errors. Populations are organized from north to south along the x-axis. At the interior loam site, data is not shown as all plants were destroyed before end of season. Populations are GCD: Grey Cloud Dunes Scientific and Natural Area; CRA: Conard Environmental Research Area; KZA: Konza Prairie Biological Station. Source locations for each population are given in Fig. 1.

Among populations, early-season survival differed significantly (Table 2) with the rank order generally consistent across sites (Fig. S2). The consistency of early-season survival among sites suggests that these differences were due to maternal provisioning which should influence germination and early-season survival equally, whereas adaptive maternal genetic effects would cause populations to differ in early-season survival among sites. Though the effect of population on the life history stages of seedpods or seed produced was not significant when early-season survival was included in the model (Table 2), lifetime seed production (i.e. overall fitness) showed patterns consistent with populations from the northern range edge being better adapted to conditions at and beyond the range edge. The northern population had greatest lifetime seed production beyond the range, and the southernmost population had the greatest seed production at the Interior-Sand site (Fig. 3).

## Discussion

Our results show that, following dispersal to the range edge or beyond, fitness of *Chamaecrista fasciculata* is dramatically reduced compared to the interior (Fig. 2), indicating that populations are unlikely to establish in this region given the conditions in the year of this study. Under conditions of reduced competition such as in this experiment, *C. fasciculata* fitness was greater on loam soils than sand soils in both the range edge and beyond regions. By contrast, *C. fasciculata* is primarily found in sand habitats at its range edge in Minnesota [34]. A likely explanation for this inconsistency is the biotic community associated with each soil type; potential competitors are both denser and taller at loam sites. In a companion study at loam sites only, we explicitly manipulated the presence of neighboring vegetation and found that neighbors both increase early-season survival and decrease seedpod production [35]. Because we removed above-ground vegetation to facilitate establishment in this study, we lessened differences in competition that likely exist between habitats. Moreover, in this study we found that early-season survival was greater on sand soils at the range edge and beyond edge sites (Fig. 2). Thus, greater early-season survival in sand habitats combined with a less competitive environment, rather than soil type alone, may underlie shifts in the habitat of *C. fasciculata* at the range edge where population persistence is already constrained by the environment. Generally, this indicates that the realized niche of a species may be restricted to sub-optimal habitats at the edge of its range as suggested by Griggs [1].

Determining whether sites beyond the range edge are demographic sinks, or simply unoccupied because of dispersal limitation, requires estimates of multiple components of fitness in these locations, especially if there are trade-offs between different life history stages [36]. We found that despite the lower survival of individuals at loam than sand sites at the range edge and beyond (Fig. 2), reproductive output per seed planted was greater at the loam than the sand sites (208% greater beyond the range, 50% greater at the range edge), perhaps because of greater nutrient or water availability. Thus, observation of multiple life history stages (e.g. survival, seedpod production) was necessary for understanding the relative contribution of each stage to population dynamics in each habitat type. Similarly, Sambatti and Rice [14] found that local adaptation of *Helianthus exilis* to serpentine and riparian sites was expressed through differential survivorship and reproductive output, respectively.

While fitness was strongly reduced at and beyond the range edge compared to the interior, our results indicate that colonists from northern range edge populations are more likely to successfully establish beyond the northern range edge than colonists from within the range (Fig. 3), even with non-adaptive differences in early-season survival among populations likely due to maternal environmental effects (Fig. S2). This is consistent with previous studies of *C. fasciculata* that have found evidence for local adaptation at geographic distances of >1000 km, including populations near the range edge [18], [37]. However, although the northern edge populations had estimates of seed production greater than one (i.e. replacement) at both sites in the beyond edge region, the 95% confidence intervals of seed production included values below one (not shown), indicating that even these populations, though best-adapted to this region, may not maintain themselves here based on data from this year of study. This is consistent with other studies that find adaptation of populations to conditions at the range edge, though absolute fitness below that necessary to sustain the populations when transplanted beyond the range edge [38], [39]. By contrast, other studies have not found a reduction in fitness beyond the range edge [40]–[42]. Thus, whether seed source matters for population establishment beyond the range edge is likely to depend on species identity, including differences in dispersal ability [43], and the abruptness of the environmental difference across the range boundary.

In conclusion, the results of this study show that soil substrate may strongly influence where colonists will establish as species shift their ranges in response to climate change, and that other aspects of the environment (e.g. competitors) that are influenced by the substrate may also play important roles in determining where populations will persist. As species are unlikely to shift their ranges synchronously, this suggests that range expansion may be limited not only by the rate at which colonists disperse, but their source, the habitats that receive them, and the competitors they encounter.

## Materials and Methods

*Chamaecrista fasciculata* (partridge pea; Fabaceae) is an annual legume native to North America that is widely distributed from central Minnesota to Massachusetts and south into Mexico [33]. It is found in prairie remnants, old fields, open woodlands, and disturbed areas on a wide variety of soil types from sand to waterlogged clay soils [32], [33]. Within the range in Illinois (the only region with extensive data), *C. fasciculata* occurs on all these soil types but most often on silty clay loam soils [32]. However, at both the northern [34] and the western range edge [33], it is found primarily on open habitats with sand soils; loam soils adjacent to all these sites are not frequently occupied by *C. fasciculata*.

In September and October 2007, we collected seed from 20 maternal plants in each of five populations at different locations, from the northern range edge in Minnesota south to Kansas and Missouri (Fig. 1, Table S1). In May and June 2008, we established common gardens in three geographic range locations: interior (central Iowa), edge (south-eastern Minnesota within ∼50 km of the furthest north known naturally occurring populations) and beyond the range (central Minnesota, approximately 120 km beyond the furthest north population recorded in the area) (Fig. 1). The locations were chosen by the availability of nearby sites with the desired soil properties in each region where we could establish common gardens. Within each region, we chose two sites differing in soil type characteristic of the habitats *C. fasciculata* occupies at the range edge (sand) and interior (loam), respectively (Table S2). Transplant sites are referred to by region and soil type (e.g. “interior – sand”, “beyond – loam”) throughout the text.

At each of the transplant sites, we planted 100 seeds from each of three source populations (CRA, GCD, KZA). Due to limited seed availability, only 52 and 24 seeds from the AFT and CUI populations were planted at most sites, no CUI seeds were planted at the beyond-loam site and no AFT or CUI seeds were planted at the edge-loam site (interior-sand N = 376; interior-loam N = 376; edge-sand N = 376; edge-loam N = 300, beyond-sand N = 376; beyond-loam N = 352). Seeds were planted in late May and early June, starting at the southern sites and moving north. Prior to planting, seeds were sterilized with 10% sodium hypochlorite (NaOCl) and scarified with a metal file. Each site was sprayed with glyphosate (Roundup®, Monsanto) at least 24 hours prior to planting, and above-ground vegetation was removed by mowing and raking to facilitate germination and lessen differences in competition among sites. Seeds were planted in a randomized block design with four blocks haphazardly placed 2 m apart at each site. In each block, seeds were planted 1 cm deep, 20 cm apart in staggered rows 17.5 cm apart, such that each plant had four neighbors 20 cm away, with pairs of rows separated by 50 cm. Rows were mowed to reduce competitors; three times at edge and beyond edge sites, and once at interior sites due to logistical constraints. There were minimal differences in vegetation between sites in the edge and beyond edge regions due to less competitive vegetation and more frequent mowing (JSG, personal observation), making the competitive environment more similar between sites in these regions. Within the range, aboveground vegetation was taller and denser at the loam site than the sand site. All sites except the beyond-loam site, due to restrictions, were fenced to exclude deer, though the fencing failed before the end of the season at the interior-loam site, preventing collection of seed pod data. In the beyond region, plants remained small and were not subject to herbivory by deer; thus differences in exposure to deer are unlikely to influence the results.

We recorded early-season survival 2–4 weeks after planting, which includes both germination and survival for the first couple of weeks. Eight weeks after planting, we recorded survival and flowering status. When plants had begun to senesce in late September and early October, we recorded survival, the number of seed pods produced by each plant, and collected at random either 10 or 10% (whichever was larger) of the seed pods on each surviving plant except at the interior-loam site where all plants were eaten or trampled by deer late in the season after the fencing was damaged. In cases where plants had senesced, it was possible to count pods that had already dehisced because the stiff pedicels with pod fragments remain attached to the plant. Pods were stored in coin envelopes at room temperature, and the average number of seeds per collected pod was recorded. Inviable or aborted seeds, judged by size and color, were not counted.

### Statistical Analyses

To model individual lifetime fitness at each site, we used aster models [30], [31] implemented in R [44]. Aster models are maximum likelihood-based linear models that allow multiple components of life history to be integrated in a single analysis, with an individual's response at each stage conditioned upon its response at the previous stage. Aster models are an improvement over previous attempts to model lifetime fitness because an appropriate distribution is specified for each life history stage, and the dependence of later life-history stages on previous stages is explicitly modeled [30], [31]. The life-history stages we modeled, and their statistical distributions, were early-season survival (*Bernoulli*), whether a plant reproduced or not (*Bernoulli*), seed pods produced (zero-truncated negative binomial), whether a plant produced any seeds (*Bernoulli*) and total seeds in sampled pods (zero-truncated negative binomial). Because seeds were counted in a subsample consisting of a random number of pods, a stage for pods sampled was included between the stages for seed pods produced and whether a plant produced any seeds (Fig. S1, node 5; see Appendix S1 for details). The stage for whether a plant produced any seeds was included to improve the fit of the model to the data, as ∼20% of plants produced pods but had no viable seeds in the sample counted. As the current aster package automatically accommodates only single-parameter exponential family distributions, the size parameters for the negative binomial distributions were chosen by fitting that distribution (fitdistr function in library MASS [45] in R) to the conditional distribution of seed pods and seeds counted. Goodness of fit for the conditional distributions of seed pods and seeds counted was assessed using Pearson residuals [46, Section 2.7] and found to have mean approximately zero and variance one with few outliers, demonstrating that these distributions appropriately model their respective stages.

### Dependence of fitness on region and habitat

To examine how region and habitat influenced individual fitness and its components, we fit an aster model with fixed effects for block, region (interior, edge, beyond), soil type (sand, loam) and the interaction between region and soil type. The current version of aster models does not allow for random effects, and as we only had four blocks per site, it is reasonable to treat them as fixed effects. The effect of each model term was tested at each life history stage. We used likelihood ratio tests to compare the fit of the full model to reduced models that sequentially dropped terms, beginning with the interaction, retaining terms that improved the model fit in subsequent tests of other terms. The block term was retained in all models. Maximum likelihood estimates of the response for each life history stage for a typical individual at each site (e.g. average block) were obtained from the final model that included all retained model terms. Variances for the parameter estimates were calculated assuming asymptotic normality of the estimates [30]. Because the estimate of seed count was obtained from a sub-sample of seed pods, it was necessary to transform the estimated number of seeds counted per plant to the total number of seeds produced per plant (see Appendix S1). For the interior-loam site, herbivory by deer prevented us from obtaining data on seed pods.

### Dependence of fitness on population

We fit aster models adding population, population × region and population × soil as fixed effects to examine differences in fitness among source populations. Maternal effects, the dependence of offspring phenotype on the maternal phenotype [47] or maternal environment (i.e. seed provisioning) often have the greatest effects at earlier life stages [48]. To statistically account for potential effects of source environment mediated by maternal phenotype or environment (hereafter, maternal environmental effects), we included in the aster model the effect of population specified at each stage of life history. The significance of population at each life history stage was then determined by using likelihood ratio tests to compare nested models as above. A significant population effect on later fitness stages when it was already included at the early-survival stage implies differences among population with respect to fitness, beyond early-expressed differences in survival, which could be influenced by maternal environmental effects. We restricted this analysis to the populations planted at all sites (CRA, GCD, KZA) as otherwise the models did not converge. Maximum likelihood estimates for each stage and total reproductive output were made for an individual from the average block from each population in each region – habitat combination.

Data and R scripts to recreate this analysis are deposited in the Dryad Repository: http://dx.doi.org/10.5061/dryad.41131ns8.

## Supporting Information

Appendix S1 Technical report for the sub-sampling of a fitness stage and transformation to estimate absolute fitness in aster models.
(DOC)

Figure S1 **Life-history stages included in aster analysis**. The distribution for each life history stage is listed below. Reproductive status is whether a plant reproduced or not, and pods sampled is the random sample of the total pods that were collected to count seeds per pod.
(TIF)

Figure S2 **Mean proportion early-season survival (± SE) for each population at each site**. Sand sites are on the left and loam sites are on the right. The interior sites are on the bottom, edge sites are in the middle and beyond edge sites are on the top. Populations are arranged from north (left) to south (right) in each plot.
(TIF)

Table S1 ***C. fasciculata*** **population source information and seeds planted at each transplant site**. Populations are organized from north to south origin. Mean annual temperature (MAT) and annual precipitation (PPT) were collected from the WorldClim data set.
(DOC)

Table S2 **Transplant site locations, and climate and soil characteristics**. Mean annual temperature (MAT) and annual precipitation (PPT) were collected from the WorldClim data set. Soil types were verified using the hydrometer method to determine the fraction of soil that was sand, silt and clay, except at CCES where soil data was already available.
(DOC)

## References

[1] Griggs RF (1914). Observations on the behavior of some species at the edges of their ranges.. Bull Torrey Bot Club.

[2] Kavanagh K, Kellman M (1986). Performance of *Tsuga canadensis* (L.) Carr. at the centre and northern edge of its range: a comparison.. J Biogeogr.

[3] Schoener TW (1975). Presence and absence of habitat shift in some widespread lizard species.. Ecol Monogr.

[4] Warren RJ (2010). An experimental test of well-described vegetation patterns across slope aspects using woodland herb transplants and manipulated abiotic drivers.. New Phytol.

[5] Werner EE, Hall DJ (1977). Competition and habitat shift in two sunfishes (Centrarchidae).. Ecology.

[6] Bazzaz FA (1991). Habitat selection in plants.. Am Nat.

[7] Andrewartha HG, Birch LC (1954). The Distribution and Abundance of Animals..

[8] Brown JH (1984). On the relationship between abundance and distribution of a species.. Am Nat.

[9] Macnair M (1987). Heavy metal tolerance in plants: A model evolutionary system.. Trends Ecol Evol.

[10] Fine PVA, Miller ZJ, Mesones I, Irazuzta S, Appel HM (2006). The growth–defense trade-off and habitat specialization by plants in Amazonian forests..

[11] Henne PD, Hu FS, Cleland DT (2007). Lake-effect snow as the dominant control of mesic-forest distribution in Michigan, USA.. J Ecol.

[12] Bissett A, Richardson AE, Baker G, Wakelin S, Thrall PH (2010). Life history determines biogeographical patterns of soil bacterial communities over multiple spatial scales.. Mol Ecol.

[13] Antonovics J, Bradshaw AD (1970). Evolution in closely adjacent plant populations. VIII. Clinal patterns at a mine boundary.. Heredity.

[14] Sambatti J, Rice KJ (2006). Local adaptation, patterns of selection, and gene flow in the Californian serpentine sunflower (*Helianthus exilis*).. Evolution.

[15] Eckhart VM, Singh I, Louthan AM, Keledjian AJ, Chu A (2010). Plant – soil water relations and species border of *Clarkia xantiana* ssp. *xantiana* (Onagraceae).. Int J Plant Sci.

[16] Hereford J (2009). A quantitative survey of local adaptation and fitness trade-offs.. Am Nat.

[17] Leimu R, Fischer M (2008). A meta-analysis of local adaptation in plants.. PLoS ONE.

[18] Etterson JR (2004). Evolutionary potential of *Chamaecrista fasciculata* in relation to climate change. I. Clinal patterns of selection along an environmental gradient in the Great Plains.. Evolution.

[19] Bischoff A, Crémieux L, Smilauerova M, Lawson CS, Mortimer SR (2006). Detecting local adaptation in widespread grassland species – the importance of scale and local plant community.. J Ecol.

[20] Turreson G (1922). The genotypic response of the plant species to habitat.. Hereditas.

[21] Clausen J, Keck D, Hiesey W (1940). Experimental studies on the nature of species. I. Effects of varied environments on western North American plants.. Carnegie Inst Wash Publ.

[22] Ehlers BK, Thompson J (2004). Do co-occurring plant species adapt to one another? The response of *Bromus erectus* to the presence of different *Thymus vulgaris* chemotypes.. Oecologia.

[23] Crémieux L, Bischoff A, Šmilauerová M, Lawson CS, Mortimer SR (2008). Potential contribution of natural enemies to patterns of local adaptation in plants.. New Phytol.

[24] Macel M, Lawson CS, Mortimer SR, Šmilauerova M, Bischoff A (2007). Climate vs. soil factors in local adaptation of two common plant species.. Ecology.

[25] Gaston KJ (2003). The Structure and Dynamics of Geographic Ranges..

[26] Sagarin RD, Gaines SD (2002). The ‘abundant centre’ distribution: to what extent is it a biogeographical rule?. Ecol Lett.

[27] Antonovics J (1976). The nature of limits to natural selection.. Annals of the Missouri Botanical Garden.

[28] Holt RD, Gomulkiewicz R (1997). How does immigration influence local adaptation? A reexamination of a familiar paradigm.. Am Nat.

[29] Lopez S, Rousset F, Shaw FH, Shaw RG, Ronce O (2009). Joint effects of inbreeding and local adaptation on the evolution of genetic load after fragmentation.. Conserv Biol.

[30] Geyer CJ, Wagenius S, Shaw RG (2007). Aster models for life history analysis.. Biometrika.

[31] Shaw RG, Geyer CJ, Wagenius S, Hangelbroek HH, Etterson JR (2008). Unifying life-history analyses for inference of fitness and population growth.. Am Nat.

[32] Foote LE, Jackobs JA (1966). Soil factors and the occurrence of partridge pea (*Cassia fasciculata* Michx.) in Illinois.. Ecology.

[33] Irwin HS, Barneby RC (1982). The American Cassiinae..

[34] Ownbey GB, Morley T (1993). Vascular Plants of Minnesota: A Checklist and Atlas..

[35] Stanton-Geddes J, Tiffin P, Shaw RG Role of climate and competitors in limiting fitness across range edges of an annual plant..

[36] Schluter D, Price TD, Rowe L (1991). Conflicting selection pressures and life history trade-offs.. Proceedings: Biological Sciences.

[37] Galloway LF, Fenster C (2000). Population differentiation in an annual legume: local adaptation.. Evolution.

[38] Geber MA, Eckhart VM (2005). Experimental studies of adaptation in *Clarkia xantiana*. II. Fitness variation across a subspecies border.. Evolution.

[39] Griffith TM, Watson MA (2006). Is evolution necessary for range expansion? Manipulating reproductive timing of a weedy annual transplanted beyond its range.. Am Nat.

[40] Marsico TD, Hellmann JJ (2009). Dispersal limitation inferred from an experimental translocation of *Lomatium* (Apiaceae) species outside their geographic ranges.. Oikos.

[41] Prince SD, Carter RN (1985). The geographical distribution of prickly lettuce (*Lactuca serriola*): III. Its performance in transplant sites beyond its distribution limit in Britain.. J Ecol.

[42] Samis KE, Eckert CG (2009). Ecological correlates of fitness across the northern geographic range limit of a Pacific Coast dune plant.. Ecology.

[43] Darling E, Samis KE, Eckert CG (2008). Increased seed dispersal potential towards geographic range limits in a Pacific coast dune plant.. New Phytol.

[44] R Development Core Team (2009). R: A Language and Environment for Statistical Computing..

[45] Venables WN, Ripley BD (2002). Modern Applied Statistics with S. New York, NY: Springer.

[46] Shaw RG, Geyer CJ, Wagenius S, Hangelbroek HH, Etterson JR (2007). Supporting data analysis for “Unifying life history analysis for inference of fitness and population growth” Technical report 658..

[47] Wolf JB, Wade MJ (2009). What are maternal effects (and what are they not)?. Philosophical Transactions of the Royal Society B: Biological Sciences.

[48] Roach D, Wulff R (1987). Maternal effects in plants.. Annu Rev Ecol Syst.

