# Supplementary material for: Interactions between Soil Habitat and Geographic Range Location Affect Plant Fitness
Source: PLoS One. 2012 May 17;7(5):e36015. doi: 10.1371/journal.pone.0036015 (PMC3355151; doi:10.1371/journal.pone.0036015)
Supplement: Appendix S1 — Technical report for the sub-sampling of a fitness stage and transformation to estimate absolute fitness in aster models. (DOC) [file pone.0036015.s001.doc]

**Appendix S1** for “Interactions between soil habitat and geographic range location affect plant fitness” by Stanton-Geddes et al.

doi:10.1371/journal.pone.0036015

**Technical report for the sub-sampling of a fitness stage and transformation to estimate absolute fitness in aster models**

Aster models allow joint analysis of multiple life history stages conditioned upon the previous stage . Ideally, all data (i.e. survival, offspring produced) is recorded at each stage, and can then be appropriately modeled in the aster analysis to give overall fitness. However, as pointed out by Shaw *et al*. , it is not always possible or practical to record all data for all life history stages, such as seeds produced, and that sub-sampling is a common practice. Shaw *et al.* show how it is possible to specify a node for the stage that is sub-sampled, and then the result for overall fitness would be proportional to the value for total fitness estimated from the aster model. It is necessary that the sub-sampling stage is distributed according to an exponential family distribution such that it can be included in the aster graph, and thus cannot be a constant value. That is, sampling of, for example, seed pods should be a percentage (i.e. 10%)of the total seed pods and not a fixed value (i.e. 10).

In the “Aster Technical Report No. 661”, Shaw *et al.* give a simulation example showing that if data is collected appropriately, sub-sampling will give appropriate estimates for total fitness with aster models. A key point is that the sub-sampling must be done with *a priori* knowledge of the requirements of aster modeling for the stage to be modeled with an exponential family distribution. The method recommended for the sub-sampling node is to choose a binomial (*n,p*) distribution, where *n* is specified by the sample size of the previous node that is being sub-sampled, and *p* is the success probability, which is determined in advance (i.e. 10% of all seed pods), but simply estimated by maximum likelihood as are the other conditional parameters. The model will estimate a different *p* in each class where *p* is known to be different (i.e. for each site, see below). The number of seed pods collected does not need to be exactly *p* for each individual, as the coefficient will simply be estimated as are the other coefficients, and thus only the expectation of seed pods sampled in each class should be equal to *p.*  For our analysis, we choose to subsample a different proportion of seed pods at each site because plants varied and size and seed pod production varied by an order of magnitude. Thus, we sub-sampled approximately 10% of seed pods on each plant at the range interior site, approximately 30% of seed pods at the range edge sites, and approximately 75% of seed pods at sites beyond the range.

When setting up the aster graphical model for this analysis, we included a “sample” node in the graph (Figure 1) that is the fraction of seed pods sampled from the total number counted (“seed pods” node). This node is fit with a Bernoulli distribution, which is in effect a Binomial(*pods, p*) distribution, where *pods* is determined by the previous node of the graph (number of seed pods) and *p* is the known fraction of pods sampled (as above). It is possible to check that the “sample” node is correctly estimated by getting the conditional mean value parameters for each stage and verifying that the estimate for “sample” reflects the known value of *p*.

Though the aster modeling is simple, difficulties arise when making estimates of total fitness for each individual, as the sub-sampling stage results in the unconditional estimates of fitness being proportional, but not equal, to true total fitness. To get true lifetime fitness, it is necessary to transform the final node by the proportion sub-sampled. For our data, to get individual plant lifetime fitness, we needed to transform the estimated average number of seeds counted per plant, to the average total number of seeds produced per plant. In this case, this is:

$$w=\frac{u_{6}*u_{3}}{u_{4}}$$

where *w* is lifetime fitness and *U* is the vector of the estimated mean value parameters from the aster model, thus *u_6_* is the maximum likelihood estimate for seeds counted (node 6 of the graph), *u_3_* is the maximum likelihood estimate for pods (node 3) and *u_4_* is the maximum likelihood estimate for pods sampled (node 4).

The calculation of standard errors and confidence intervals for this transformation requires using the delta method to get the asymptotic variance – covariance matrix for *w*.

To do this, we calculated the Jacobian matrix for the transformation of *u_i_* to *w*:

$$J=\left(\frac{u_{6}}{u_{4}},\frac{-u_{6}u_{3}}{u_{4}^{2}},\frac{u_{3}}{u_{4}}\right)$$

and then we calculated the asymptotic variance – covariance matrix (*M_u_*)for *U* using the component gradient,which is $\partial U/\partial w$, from the output of the predict.aster function and the component fisher, which is the expected Fisher information, from output of the aster function (in this example, called “model”) follows:

$$M_{u}=\frac{\partial U}{\partial w}\times(fisher)^{-1}\times(\frac{\partial U}{\partial w})^{T}$$

In R, this is written as:

*M_u_* <- gradient %*% solve(model$fisher) %*% t(gradient)

Then, by the delta method, the asymptotic variance – covariance matrix for *w* is:

*M_w_* <- *J * M_u_ * J*^T^

and the diagonal elements of this matrix give the standard errors for *w.* Code for this was modified from .

*Data accessibility*

Data and R scripts necessary to repeat this analysis are deposited in the Dryad Repository: http://dx.doi.org/10.5061/dryad.41131ns8

Figure 1. Graphical model used in aster analysis.


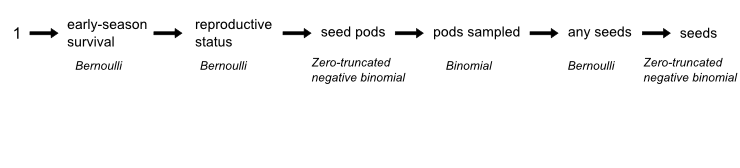


**References:**
