# Supplementary figures and images for: Interactions between Soil Habitat and Geographic Range Location Affect Plant Fitness
Source: PLoS One. 2012 May 17;7(5):e36015. doi: 10.1371/journal.pone.0036015 (PMC3355151; doi:10.1371/journal.pone.0036015)

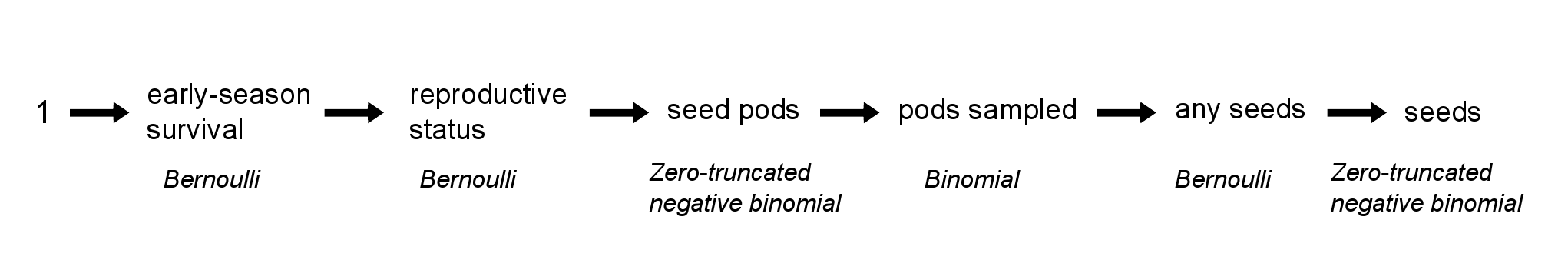

Supplement: Figure S1 — Life-history stages included in aster analysis. The distribution for each life history stage is listed below. Reproductive status is whether a plant reproduced or not, and pods sampled is the random sample of the total pods that were collected to count seeds per pod. (TIF) [file pone.0036015.s002.tif]

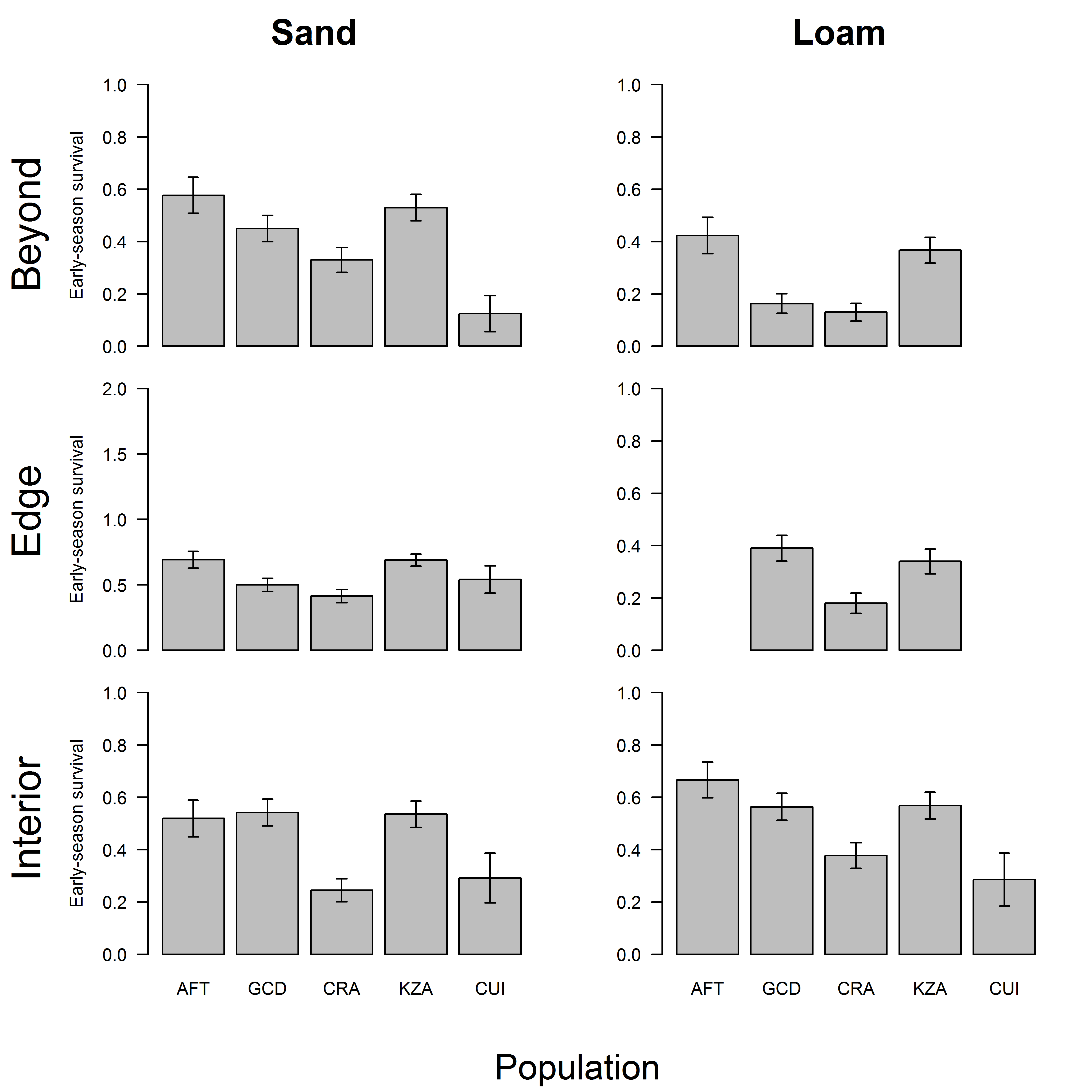

Supplement: Figure S2 — Mean proportion early-season survival (± SE) for each population at each site. Sand sites are on the left and loam sites are on the right. The interior sites are on the bottom, edge sites are in the middle and beyond edge sites are on the top. Populations are arranged from north (left) to south (right) in each plot. (TIF) [file pone.0036015.s003.tif]
