# Supplementary material for: Interactions between Soil Habitat and Geographic Range Location Affect Plant Fitness
Source: PLoS One. 2012 May 17;7(5):e36015. doi: 10.1371/journal.pone.0036015 (PMC3355151; doi:10.1371/journal.pone.0036015)
Supplement: Table S1 — C. fasciculata population source information and seeds planted at each transplant site. Populations are organized from north to south origin. Mean annual temperature (MAT) and annual precipitation (PPT) were collected from the WorldClim data set. (DOC) [file pone.0036015.s004.doc]

**Table S1. *C. fasciculata* population source information and seeds planted at each transplant site.** Populations are organized from north to south origin. Mean annual temperature (MAT) and annual precipitation (PPT) were collected from the WorldClim data.

**Population Location Lat/Long Region MAT (°C) PPT (mm) Soil type**

AFT Afton State Park, Afton, MN 44°51’53N Edge 7.0 778 Sand

92°46’21W

GCD Grey Cloud Dunes Scientific and 44°47’19N Edge 7.5 754 Sand

Natural Area, Cottage Grove, MN 92°57’29W

CRA Conard Environmental Research Area 41°41’14N Interior 8.9 882 Loam

(Grinnell College), Kellogg, IA 92°52’16W

KZA Konza Prairie Biological Station, 39°07’07N West edge 11.9 876 Loam

(KSU), Manhattan, KS 96°32’15W

CUI Cuivre River State Park, Troy, MO 39°01’00N Interior 12.3 963 Loam

90°55’16W
