# Supplementary material for: Interactions between Soil Habitat and Geographic Range Location Affect Plant Fitness
Source: PLoS One. 2012 May 17;7(5):e36015. doi: 10.1371/journal.pone.0036015 (PMC3355151; doi:10.1371/journal.pone.0036015)
Supplement: Table S2 — Transplant site locations, and climate and soil characteristics. Mean annual temperature (MAT) and annual precipitation (PPT) were collected from the WorldClim data set. Soil types were verified using the hydrometer method to determine the fraction of soil that was sand, silt and clay, except at CCES where soil data was already available. (DOC) [file pone.0036015.s005.doc]

**Table S2. Transplant site locations, and climate and soil characteristics**. Mean annual temperature (MAT) and annual precipitation (PPT) were collected from the WorldClim data set. Soil types were verified using the hydrometer method to determine the fraction of soil that was sand, silt and clay, except at CCES where soil data was already available.

**Site Location Lat/Long Region Soil MAT (°C) PPT (mm) % sand % clay**

Beyond - Loam Runestone County Park, Kensington, MN 45°48’43N Beyond Loam 5.2 635 38 33

95°39’55W

Beyond - Sand Lake Ida, Douglas County, MN 45°57’05N Beyond Sand 5.2 596 71 11

95°25’31W

Edge - Loam St. Croix Watershed Research Station, 45°10’04N Edge Loam 6.8 774 68 29

Marine-on-St. Croix, MN 92°45’53W

Edge - Sand Cedar Creek Ecosystem Science Reserve, 45°24’10N Edge Sand 6.3 751 94 1
 Bethel, MN 93°11’28W

Interior - Loam Conard Environmental Research Area 41°41’03N Interior Loam 8.9 882 33 14

(Grinnell College), Kellogg, IA 92°51’42W

Interior - Sand Iowa River Conservation Area, 42°04’31N Interior Sand 8.4 854 83 6

Marshalltown,IA 92°57’02W
